# Theory of Hermaphroditism

**Published:** 1844-02-24

**Authors:** 


### THEORY OF HERMAPHRODITISM.

Dr. Knox considers that as in vertibrate animals the organs of both bronchial and pulmonary respiration are found uniformly to co-exist at an early period, either kind becoming subsequently developed at the expense of the other, so as to the generative organs the type is originally double, the organs of both sexes co-existing in the early stages of development, but one set alone in general developing afterwards at the expense of the antagonist set.

